# Plant-Based Diets versus the Mediterranean Dietary Pattern and Their Socio-Demographic Determinants in the Spanish Population: Influence on Health and Lifestyle Habits

**DOI:** 10.3390/nu16091278

**Published:** 2024-04-25

**Authors:** Elena Sandri, Marco Sguanci, Eva Cantín Larumbe, Germán Cerdá Olmedo, Lisa Ursula Werner, Michela Piredda, Stefano Mancin

**Affiliations:** 1 Faculty of Medicine and Health Sciences, Catholic University of Valencia San Vicente Mártir, c/Quevedo, 2, 46001 Valencia, Spain; elena.sandri@ucv.es (E.S.); german.cerda@ucv.es (G.C.O.); 2 Doctoral School, Catholic University of Valencia San Vicente Mártir, c/Quevedo, 2, 46001 Valencia, Spain; 3 Research Unit Nursing Science, Campus Bio-Medico di Roma University, Via Alvaro del Portillo, 21, 00128 Rome, Italy; marco.sguanci@unicampus.it; 4 Faculty of Data Science, Polytechnical University of Valencia, Camí de Vera, s/n, 46022 Valencia, Spain; evacantinlarumbe@gmail.com; 5 Faculty of Teaching and Science of Education, Catholic University of Valencia San Vicente Mártir, c/Quevedo, 2, 46001 Valencia, Spain; lu.werner@ucv.es; 6 Department of Biomedicine and Prevention, University of Rome “Tor Vergata”, Viale Montpellier, 1, 00128 Rome, Italy; stefano.mancin.84@students.uniroma2.eu

**Keywords:** vegetarian diet, vegan diet, Mediterranean diet, healthy lifestyle, survey, Spain

## Abstract

Background: Plant-based diets are becoming more and more widespread among the Spanish population, progressively replacing the Mediterranean dietary pattern. Different studies have shown the motivations for adherence to these diets, and others have highlighted some health advantages and disadvantages. Purpose of the study: Further studies are needed to define the socio-demographic determinants that influence the choice of a plant-based diet and to study the relationship that the choice of dietary pattern has on the health and lifestyle habits of the population. Methods: A descriptive, cross-sectional study was conducted on the Spanish population. The NutSo-HH questionnaire, developed and validated by the research team, was used to gather socio-demographic, nutritional, social, and lifestyle information through non-probabilistic snowball sampling. Results: The questionnaire was completed by 22,181 Spanish citizens, of whom only 19,211 were of interest to the study. The socio-demographic variables gender, age, educational level, income level, and place of residence do not seem to influence the prevalence of a plant-based diet (*n =* 1638) compared to a Mediterranean diet (*n =* 17,573). People following a vegetarian or vegan diet have a lower BMI, and they consume less fried food, fast food, and ultra-processed dishes and fewer energy drinks or sugary beverages. They also do more exercise and sleep longer hours, smoke less, and consume alcohol less frequently. However, there seem to be more diagnosed eating disorders among people who follow a plant-based diet than those who follow a Mediterranean diet. Conclusions: People who adopt a plant-based diet tend to exhibit healthier lifestyle patterns and consume fewer foods that are detrimental to their health. However, it is essential for such dietary choices to be supervised by healthcare professionals to mitigate the risk of maladaptive behaviors evolving into eating disorders.

## 1. Introduction

Spain is recognized as a typical Mediterranean country, and for centuries, the Mediterranean diet has been the commonly adopted dietary pattern in the country [1]. The Mediterranean diet is characterized by a high consumption of minimally processed plant foods, such as fruits, vegetables, nuts, seeds, legumes, and whole grains. The predominant use of extra virgin olive oil as the main source of fat and the moderate consumption of dairy products, along with a variety of herbs and spices as seasonings, contributes to the diversity and richness of flavors in Mediterranean dishes. The consumption of fish, especially fish rich in omega-3 fatty acids, is recommended two to three times a week, while the consumption of red meat, processed meat, and foods high in saturated fats and added sugars is strongly limited. Wine, especially red wine, is consumed in moderation and preferably during meals, and it is considered an integral part of the Mediterranean culinary experience [2].

The health benefits of the Mediterranean diet have been extensively studied and documented in the scientific literature, and they have been associated with a longer life expectancy and a better overall quality of life [3,4,5,6,7]. Despite its proven benefits, it seems that in Western countries, and particularly in Spain, the Mediterranean diet is gradually being abandoned [8]. In recent years, there has been a decline in the interest among the Spanish population in the Mediterranean diet, with growing attention to other diets, such as intermittent fasting, the ketogenic diet, or a plant-based diet [9]. More and more people are adopting a plant-based diet (PBD) in all its variations [10]. The prevalence of PBD in Europe varies significantly among countries and regions, making it difficult to establish an exact percentage. Estimates typically range from 1% to 10% [11], but even these figures can vary based on cultural attitudes towards vegetarianism and veganism, the availability of vegan products, dietary habits, and socioeconomic factors [12]. In contemporary society, a growing consensus is seen among individuals, organizations, and scientists that reducing meat consumption can have positive impacts on various aspects, such as animal welfare, sustainability, and human health [13]. Behind the spread of PBDs, various motivations can be found, including the desire to improve animal welfare by reducing meat consumption [14]. By opting for plant-based diets or reducing meat consumption, people can contribute to reducing the demand for products from intensive livestock farming, potentially improving the treatment of animals. Moreover, a predominantly plant-based diet reduces the environmental impact, as PBDs tend to have a smaller ecological footprint than diets rich in animal products [15,16]. Finally, proponents of a plant-based diet argue that it has beneficial effects on human health [17]. Numerous studies have linked excessive meat consumption, especially red and processed meats, to health problems, such as heart disease [18,19], certain types of cancer [20], obesity [21], and type 2 diabetes [22]. By moderating meat consumption and incorporating more fruits, vegetables, and whole grains into the diet, people can reduce the risk of developing these health problems and improve their overall well-being. To date, there is no universally accepted definition of the term “plant-based diet”. In some cases, it has been used to refer to omnivorous diets with a low content of foods of animal origin only [23]. The main categories of diets we refer to in this research are as follows: flexitarian or semi-vegetarian diets (FDs), which are mainly vegetarian with the occasional inclusion of meat or fish; vegetarian diets (VDs) (lacto-ovo-vegetarian: plant-based except for dairy products and/or eggs), and vegan diets (VGs) (100% plant-based).

In the current research landscape, there is a significant gap regarding plant-based diets. Although considerable attention has been paid to understanding the motivations behind adopting vegetarian and vegan lifestyles [24], there is a lack of studies accurately quantifying the number of individuals adhering to these dietary patterns, studying their relationship with socio-demographic determinants, and exploring the relationship with other lifestyle habits. A recent study [25] analyzing the variation in the prevalence of PBDs in the Spanish population between 2001 and 2017 and its association with lifestyle habits and health variables explored only four variables, namely physical activity, tobacco consumption, alcohol consumption, and body mass index (BMI) [25].

### Study Objective

In order to integrate the available literature on the consumption of plant-based diets among the Spanish population, this study aimed to investigate the prevalence of vegetarian, vegan, and flexitarian dietary patterns compared to the Mediterranean dietary pattern in the Spanish population, to explore whether the adoption of a specific diet is influenced by socio-demographic factors (such as age, sex, level of education or income, size of town of residence, or size of region of residence), and to describe the relationship between a type of diet and social/lifestyle habits adopted in the population.

## 2. Materials and Methods

### 2.1. Study Design and Sampling

A descriptive, cross-sectional survey was conducted with adults (over 18 years of age) residing in Spain. Participants were excluded from the study if, at the time of survey completion, they had any medical condition or constraint that could impact their dietary patterns, including hospitalization or confinement. 

### 2.2. Ethical Approval

This research adheres to the STROBE Guidelines [26] (Supplementary Materials). Approval for the study was obtained from the Research Ethics Committee of the Catholic University of Valencia under approval code UCV/2019-2020/152. Informed consent was obtained from all participants prior to their involvement in the study, and the ethical recommendations contained in the Declaration of Helsinki [27] were always followed. 

### 2.3. Instrument

A self-designed questionnaire was utilized for data collection, comprising several sections: a diet section addressing the type of diet followed, the consumption of various food groups, and drinking habits; a section exploring the presence of diagnosed eating disorders or their symptoms; a section on physical activity and lifestyle habits relevant to health; and finally, anthropometric data such as weight, height, and socio-demographic variables. The development and psychometric testing of the instrument adhered to rigorous methodological standards and involved an expert panel, including a nutritionist, two family physicians, two psychologists, a social educator, and a communication specialist. The validation of the questionnaire included a pilot study with 53 individuals whose characteristics were similar to the target population. 

### 2.4. Data Collection

The questionnaire was distributed utilizing non-probabilistic “snowball” sampling [28]. A Google Forms survey was created for this purpose and disseminated primarily through online channels, through which the people who helped with the dissemination shared the link to the questionnaire. Initially, the Instagram account @elretonutricional was utilized, along with the personal social networks of the researchers through WhatsApp, LinkedIn, Facebook, and Twitter and email distributions to various associations. Additionally, efforts were made to physically disseminate the questionnaire to accommodate individuals less inclined toward digital platforms. This involved posting flyers in various shops and businesses frequented by individuals with diverse socio-demographic characteristics. The data collection spanned from August 2020 to November 2021.

### 2.5. Variables

For nutritional analysis, we opted to utilize the IASE index (Índice de Alimentación Saludable para la población Española) [29], which was modified to create a condensed version. This adapted index assigns a maximum score of 73 points, aligning with the level of adherence to the recommendations outlined by the Spanish Society of Community Nutrition (SENC) [30]. Based on the IASE score obtained, the dietary habits of the population can be classified into three categories: 58.4 < IASE < 73: “Healthy”; 36.5 < IASE < 58.4: “Needs changes”; IASE < 36.5: “Unhealthy”. Table 1 illustrates the categorization of variables utilized for the IASE.

The socio-demographic variables considered were categorized as follows:

- Sex: analyzed in binary way; male and female.
- Age: categorized into four groups; young individuals (18–25 years old), young adults (26–45 years old), adults (46–65 years old), and older adults (over 65 years old).
- Level of education: basic studies (no studies, primary or secondary education, vocational training, or baccalaureate) and higher studies (bachelor’s, master’s, and PhD).
- Income level: low income (<2200 EUR/month per household) and medium–high income (>2200 EUR/month).
- Municipality: small municipalities (<2000 inhabitants), medium-sized towns (between 2000 and 10,000 inhabitants), and cities with more than 10,000 inhabitants.
- Place of residence: based on the different regions of Spain.

Nutritional and health habit variables not addressed in the IASE were categorized on a 4-point Likert scale (from 1 = none or low frequency to 4 = maximum frequency), while variables related to eating disorders were categorized on a 6-point Likert scale (Table 2). Exceptions were the body mass index (BMI) and minutes of exercise, which were used as numerical variables.

### 2.6. Data Analysis

Data were checked to identify any erroneous entries or outliers, and extreme BMI values (<14 and >40) were excluded. Descriptive and inferential statistical analyses were performed. Discrete variables were presented as absolute values and percentages, while continuous variables were expressed as means and standard deviations. The normality of the data was evaluated using the Lilliefors Test (Kolmogorov–Smirnov) at a 95% significance level [31], revealing a non-normal distribution. Consequently, Chi-2, Mann–Whitney, and Kruskal–Wallis tests [32], which are non-parametric, were employed to compare differences between categories of variables. After applying Kruskal–Wallis test, a post hoc analysis was conducted with Bonferroni correction to study pairwise comparisons. All the data analysis was carried out with Python 3.9.13 [33].

## 3. Results

**Figure 1:**
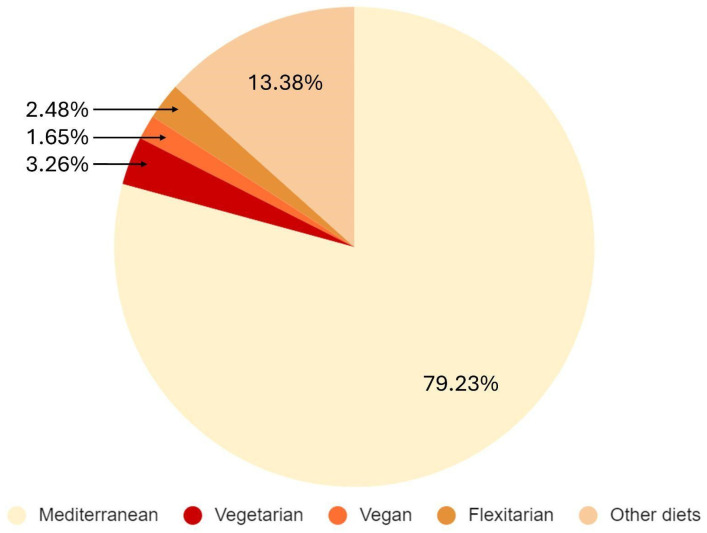
Distribution of the types of diet adopted (*n =* 22,181).

From the total population (*n =* 22,181), 17,573 individuals (79.23%) reported following a Mediterranean dietary pattern, 723 (3.26%) followed a vegetarian diet, 365 (1.65%) chose a vegan diet, 550 (2.48%) defined themselves as flexitarians, and the remaining 13.38% (*n =* 2970) reported other types of diets (Figure 1).

For the purpose of the study, individuals who reported following diets other than Mediterranean and plant-based diets were not included in the data analysis. Therefore, the final valid sample consisted of 19,211 individuals. Details of the sample’s socio-demographic characteristics are described in Table 3.

The distribution of the sample’s dietary behaviors according to socio-demographic variables, including gender, age, educational level, income level, and municipality of residence, showed no statistically significant differences among the analyzed groups. These findings suggest that these socio-demographic factors did not exert an influence on dietary preferences (Table 4).

**Figure 2:**
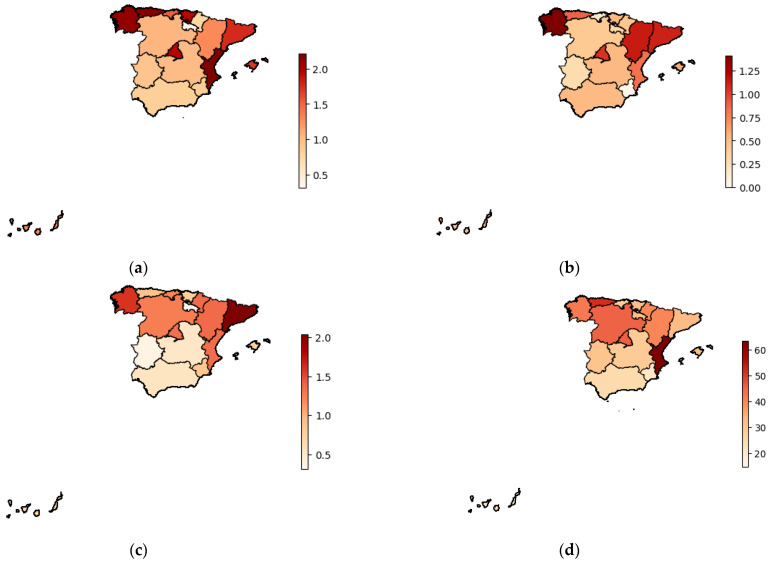
The prevalence of different diets in the Spanish regions: (**a**) the prevalence of vegetarianism; (**b**) the prevalence of veganism; (**c**) the prevalence of flexitarianism; and (**d**) the prevalence of the Mediterranean diet.

The prevalence of the different diets in the Spanish regions (Figure 2a–d) was calculated by dividing the number of people following each type of diet in a region by the number of inhabitants in each region. The data reported by the National Institute of Statistics (INE) were used for this purpose. No statistically significant differences were found in the prevalence of any type of diet depending on the region (vegetarian diet *p =* 0.45; veganism *p =* 0.39; flexitarian diet *p =* 0.42; and Mediterranean diet *p =* 0.36). 

Subsequently, the relationships between the four different dietary choices considered—Mediterranean, vegetarian, vegan, and flexitarian—and various health-related variables or social and lifestyle habits were explored. Statistically significant differences were observed among the groups regarding variables such as BMI (*p* < 0.001), the healthy eating index (*p* < 0.001), and unhealthy food consumption (fried food: *p* < 0.001; fast food: *p* < 0.001; and ultra-processed food: *p* < 0.001). Significant differences were also found in beverage consumption, including the frequency of sugary drink intake (*p* < 0.001) and coffee and energy drink consumption (*p* < 0.001). However, no significant differences were noted in water consumption except between the Mediterranean and Vegan diet groups (*p* < 0.001), and significant differences in fruit juice consumption were found between the Mediterranean and flexitarian groups (*p* < 0.001). Sedentary behavior did not appear to influence dietary choices, while differences in perceived health levels were significant only between the Mediterranean and vegan diet groups (*p =* 0.02), with the latter generally reporting feeling better. Regarding physical activity, differences were observed among all groups, with individuals following a plant-based diet, in all its variations, engaging in significantly more sport compared to those following a Mediterranean diet (*p* < 0.001). Regarding sleep habits, no differences were found among individuals adopting different dietary patterns in terms of sleep quality or perception, but significant differences were observed in sleep hours between those following a Mediterranean diet, vegetarians, and vegans, with the latter two groups seeming to sleep more (M versus VD: *p* < 0.001; M versus VG: *p* < 0.001). Finally, when social habits were analyzed, significant differences emerged for almost all variables: smoking (*p* < 0.001), alcohol consumption (*p* < 0.001), and nightlife (*p* < 0.001), with a tendency to adopt healthier habits for those following a plant-based diet. On the other hand, with respect to the eating disorder variables, which also showed significant differences (*p* < 0.001), these behaviors seemed to be more frequent among vegetarians and vegans than among those who follow a Mediterranean diet (Table 5). (For more detailed comparisons between the habit variables and the different dietary styles analysed, see the figures reporting the results of Dunn’s tests in Appendix A).

## 4. Discussion

This study aimed to describe the prevalence of vegetarian, vegan, and flexitarian dietary patterns compared to the Mediterranean dietary pattern, as well as the relationship between such diets and socio-demographic factors and social/lifestyle habits in the Spanish population.

The majority of the sample (79.23%) followed a Mediterranean dietary pattern. Such a prevalence is expected for people living in a typically Mediterranean country such as Spain, which, for decades, has traditionally followed a predominantly Mediterranean diet [34]. Although the Mediterranean diet is still the predominant diet on the peninsula, more than 20% of those surveyed reported currently following another type of diet. This confirms the trend found in studies that have evidenced that Spaniards are progressively moving away from the Mediterranean dietary pattern [8,35].

Regarding the prevalence of a plant-based diet, 7.39% of the respondents stated that they chose this type of diet; more specifically, 3.26% defined themselves as vegetarians, 1.65% as vegans, and 2.48% as flexitarians. Again, these data align with those found in the literature for the European population [11], although the prevalence of veganism appears to be slightly lower than that found in a recent study [36]. Moreover, the percentage of people choosing a vegan diet is still lower than the percentage of those choosing a flexitarian or vegetarian diet, in line with previous studies [37]. It can be hypothesized that this percentage will be reversed in a few years, as several studies in Europe indicate that adherence to the vegan diet is increasing compared to adherence to the less restrictive plant-based dietary options [25,38]. There may be several reasons to explain the increasing trend of the vegan dietary pattern among the Spanish population. Two strong reasons are environmental concerns and animal welfare awareness, which are both of great interest in Western countries at present [13,14]. The vegan diet, due to its characteristics and the products it includes, reduces the environmental impact and the consumption of animals, which makes it a valid option for people who are sensitive to these issues [15,16]. Moreover, the greater availability of information on this type of diet on the internet and in networks [39], as well as the increased acceptance of vegan products among the population in recent years [40], could also favor the spread of this dietary style.

When attention was focused on the relationship between socio-demographic variables and the type of diet adopted, no significant differences were found for any of the groups explored in our sample. This suggests that the choice of one dietary style or another in Spain is not influenced by gender, age, level of education or income, or the area where a person resides. There are several possible hypotheses to explain this behavior. 

On the one hand, we could highlight the democratization of health information, which may have partly leveled out the differences between population groups. In recent decades, the use of information and communication technologies (ICTs) has spread progressively in society [41]. One of the fields witnessing remarkable developments in the use of ICTs is healthcare, particularly through the emergence of eHealth. Mobile health has expanded the provision of health services via smartphones and the development of specific mobile apps, offering convenient and immediate access to a vast number of people [42,43]. Also, the increasing use of social media, which brings people from all locations and cultures into contact with each other, means that the same food habits can be adopted via imitation in very different environments [44,45]. Finally, the abundant publication on these channels of information concerning nutrition, cooking, or gastronomy can invite people to put aside more classical nutritional habits to try new types of diets, new products, or different recipes [46,47].

Another possible explanation for the lack of dependence found between dietary pattern choice and socio-demographic determinants could be that, in recent decades, particularly in Western countries, there has been a significant change in attitudes towards food and health in general. This has led to a greater awareness of different types of diets and a more widespread adoption of healthy lifestyles across the population, regardless of gender, age, educational level, income level, or place of residence [48,49].

Finally, a possible explanation can also be found in the availability and accessibility of food, which may not vary significantly between different socio-demographic groups in Spain. Industrialization and globalization have meant that, in developed countries, the range of foodstuffs on offer is very wide, and different types of products and brands can be found in a wide variety of establishments, ranging from neighborhood grocery stores to large supermarkets [50,51]. This means that almost all the population has easy and affordable access to a large variety of foods, especially basic ingredients such as fruits, vegetables, and olive oil, which are fundamental to the Mediterranean diet and to many vegetarian and vegan diets [52]. It is also worth noting that the increase in demand has led to a decrease in the cost of these products. This makes them affordable for a larger number of people, including citizens with lower incomes.

When critically analyzing the results obtained for the health and lifestyle variables with respect to the different types of diet (Table 5), regarding the dimension linked to movement and sport habits, no statistically significant differences were observed in terms of sedentary behavior. Where the differences become important is in the time devoted to sport. In the case of people who follow a Mediterranean diet, the average time spent doing sport per week was 145 min, which is slightly below the 150 min recommended by the WHO in terms of health [53]. The average weekly time spent on sports is 40–45 min more for vegetarians and flexitarians than for people who follow the Mediterranean diet, and in the case of vegans, it is even 70 min more, rising to 213 min. To determine whether there was a statistically significant difference between the followers of a plant-based diet and those who adopted a Mediterranean diet, these two groups were analyzed with the Mann–Whitney U test, which obtained a *p*-value < 0.001. Regular physical activity has long been proven to offer a wide range of physical, mental, and emotional health benefits, making it an important part of a healthy lifestyle. Physical activity helps burn calories and maintain a healthy weight [54]. It also contributes to fat loss and the development of lean muscle mass [55]. This strengthening of muscles and bones helps prevent muscle wasting and osteoporosis [56]. Physical exercise also strengthens the heart and improves blood circulation, thus reducing the risk of heart disease [57]. Mental and emotional health is also influenced by sport. Regular physical activity can improve cognitive function and reduce the risk of developing neurodegenerative diseases [58]. It can also improve sleep quality and help combat insomnia [59]. In addition, physical exercise releases endorphins, neurotransmitters that help reduce stress, anxiety, and depression. Achieving sporting accomplishments or simply beating one’s earlier achievements in training also promotes a sense of well-being and increases self-esteem [60].

Certainly, the relationship between diet and physical activity can vary widely between individuals, and the amount of physical exercise a person undertakes may depend on a variety of factors, including their lifestyle, personal preferences, previous level of physical activity, and health goals. The results found do not allow for the establishment of a general rule that people following a vegetarian or vegan diet do more sport than those following a Mediterranean diet. However, we could hypothesize some reasons for the tendency found. Firstly, vegetarian and vegan diets are often associated with greater attention to personal well-being and to the connection between food and health. This may lead these individuals to prioritize exercise as another way to improve their health and quality of life [61]. Secondly, many people adopt a PBD for ethical reasons, such as concerns for the environment, sustainability, or animal rights. These same values could extend to taking care of one’s own body, which could include regular exercise [62]. Last but certainly not least, it is worth noting that generally, people who follow a vegetarian or vegan diet often connect with a community that shares their values and goals. This community can provide support and motivation to maintain an active and healthy lifestyle, including regular exercise [63].

The difference in BMI between people who follow a traditional Mediterranean diet and those who follow a plant-based diet also stands out. In the former, the BMI is almost one and a half points higher than in the latter. These data are consistent with previous studies that have highlighted that people who follow a plant-based diet tend to have a lower body mass index (BMI) compared to those who do not. In addition, adopting a PBD also appears to be effective for weight loss [10,64]. Weight loss in people following a plant-based diet (PBD) can be attributed to the fact that, compared to the traditional Mediterranean pattern, the PBD usually involves a higher intake of fiber, polyunsaturated fats, and plant proteins, as well as a lower intake of energy, saturated fats, and animal proteins. These changes in dietary composition may contribute to more effective weight reduction in those who adopt a PBD [10]. The lower mean BMI found among the population following a plant-based diet could also be partly attributed to the fact that these people, in our sample, were more physically active than those following a Mediterranean diet. It has been shown that regular physical activity can help control weight gain or even promote weight loss [54,55].

With respect to the healthy nutrition index for the Spanish population (IASE), statistically significant differences were observed between all groups. The IASE score was lower for plant-based diets than for the Mediterranean diet and especially low (up to 13 points lower) for the vegan diet. This difference in the IASE score is explained by the fact that the IASE score is calculated on the basis of the consumption of a varied diet that includes several food families, including animal proteins such as meat and dairy products [29]. A vegan diet excludes the consumption of these foods, and therefore, the IASE score is much lower in this specific case; for this reason, the IASE score is not useful in giving us reliable information about the goodness of the diet followed. 

The variables concerning the consumption of fried foods, the consumption of fast food, and the consumption of ultra-processed foods show that respondents who indicated that they followed a plant-based diet seemed to adopt healthier nutritional habits than those who followed the Mediterranean diet. Numerous studies have illustrated the detrimental health effects of fried food and ultra-processed foods [65,66], as they are often loaded with unhealthy fats, added sugars, sodium, and artificial additives while lacking essential nutrients [67]. Fried foods can pose a particular health risk. They are often high in trans fats, saturated fats, and calories. Their regular consumption has been associated with an increased risk of obesity and elevated cholesterol levels, as well as hypertension and an elevated risk of cardiovascular disease [68,69]. The regular consumption of fast food has been correlated with an increased risk of obesity, type 2 diabetes, heart disease, and certain cancers. These foods often lack the vitamins, minerals, and dietary fiber necessary for optimal health [70,71]. Ultra-processed foods often contain high levels of refined carbohydrates, unhealthy fats, added sugars, and artificial additives. The repeated consumption of such foods has been linked to weight gain, metabolic disorders, increased inflammation, and a higher incidence of chronic diseases such as obesity, type 2 diabetes, cardiovascular disease, and certain cancers [66,72].

As regards self-perceived levels of health, it seems that vegans have a more positive perception of their state of health compared to people who follow a Mediterranean diet, although the difference is not very high. 

Finally, another dimension in which significant differences were found was social habits, with people following a plant-based diet in general smoking less, consuming alcohol less frequently, and tending to go out less frequently to parties and for entertainment than people following a traditional diet. These social habits are associated with a healthy lifestyle, and so the reasons that might underlie the trend found are similar to those discussed in the case of physical activity. As explained, people who follow a PBD often have a greater awareness of the impact of their habits on health [61]. Therefore, they may prioritize their personal well-being over the pursuit of momentary pleasures such as smoking, excessive drinking, or engaging in partying and fun activities. They opt for activities that promote long-term physical, mental, and emotional health. The ethical values of concern for animal welfare and the environment that normally underpin the personal choice to adopt a plant-based diet also play a role [62]. These same values can extend to other aspects of their lives, including the choice to avoid tobacco and alcohol, which can be detrimental to health and the environment.

Although most of the results show that a plant-based diet seems healthier than the Mediterranean one, we must underlie the strong and countless evidence of the benefits of the Mediterranean diet model for health and longevity. The effects of the Mediterranean diet on the prevention and treatment of cardiovascular disease are well-recognized in numerous studies [73,74], as are the positive effects on metabolic disorders [75] and some types of cancer [76].

Moreover, it is necessary to remember that a strict plant-based diet entails potential health risks. In a recent review, its correlation with the onset of physical disorders was described [77] due to a low intake of and/or a risk of deficiencies in specific nutrients that can influence bodily and mental functions. A strong incidence of mental health problems has been found in the vegan population compared to other types of diet, contributing to a lower quality of life [78,79].

Given the very limiting characteristics of this nutritional model, it is essential to consult a dietitian or nutrition professional regarding one’s nutritional status and any symptoms that may appear with the aim of promptly preventing nutritional inadequacies and minimizing any negative health consequences.

### Strengths and Limitations

One of the weaknesses of the study lies in the sampling technique used and in the prevalent dissemination through social media. Snowball sampling offers the advantage of reaching a large number of responses, but it may introduce a self-selection bias, as the sample collected depends on initial participants recommending other potential participants. Disseminating the survey through social media, with the support of several influential people and professionals in these fields, led to a considerable number of respondents showing an interest in nutrition and health. It is reasonable to assume that these people have above-average knowledge on the subject and that they usually strive to adopt healthier habits. An important effort was made to try to mitigate self-selection bias by also conducting a physical dissemination of the survey. To this end, several establishments throughout Spain with a diverse clientele (such as pharmacies, tobacconists, associations of citizens, etc.) were involved. Their owners were provided information about the project and asked to display posters on their premises with a QR code linking to the questionnaire so that customers in these establishments could fill out the survey if they wished to do so. 

Gender bias was also observed, as about 80% of the respondents were women. There is a greater predisposition in the female public, also observed in other studies [80,81,82,83], to collaborate with these types of studies, and perhaps there is also a greater concern and sensitivity to nutrition and health issues among women than among their male counterparts. This notwithstanding, due to an awareness of the importance of achieving a balanced sample, an additional effort was made to reach more men as participants in the study, finally achieving a sample of 3682 men.

The dissemination of the sample via social networks could have introduced age-related and educational biases in the sample, leading to a greater participation rate among younger individuals and people with a higher level of education. Typically, younger people are more inclined towards and engaged with social media platforms, allocating more time to their utilization, thus enhancing their likelihood of completing the survey. A higher level of education also makes people more likely to be concerned about health and to seek information about it in various media, including social media. Our sample, with a mean age of 34 years and 68.51% of respondents with a higher education level, partially suffered from these biases. We are currently carrying out a second dissemination of the survey, trying to collect a larger sample of older, less educated, and more male respondents.

Another limitation is that the IASE healthy nutrition index is based on a varied diet comprising all food groups, including meat and meat products. For a plant-based diet and, in particular, for a vegan diet, the IASE cannot be chosen as a stand-alone indicator of the goodness of nutrition. It needs to be included in an overall picture of the consumption of other food groups and dietary habits, as we have tried to do in this study.

The undoubted strengths of the study are the very large sample size (*n =* 19,211), variety, and heterogeneity, as adults of all ages and educational and income levels were represented. Geographical variety was also guaranteed, as all regions of Spain were significantly represented in the sample. This made it possible to obtain reliable information on the health status and nutritional and lifestyle habits of the entire Spanish population.

## 5. Conclusions

People who choose to adopt a plant-based diet not only demonstrate healthier lifestyle patterns but also tend to consume a wider variety of nutrient-rich and antioxidant-rich foods. This type of diet is often associated with numerous health benefits, including a lower risk of cardiovascular diseases, obesity, and some forms of cancer. However, it is important to recognize that following a plant-based diet requires adequate planning to ensure the intake of all essential nutrients, such as proteins, vitamins, and minerals. Furthermore, it is crucial to emphasize that plant-based diets are not necessarily suitable for everyone and may require careful monitoring, especially in the initial stages of adoption. Supervision by healthcare professionals is essential to ensure that individuals maintain an optimal nutritional balance and to prevent any deficiencies or imbalances that may result from improperly planned diets. 

A plant-based diet should be strictly monitored in more vulnerable groups. For instance, newborns or pregnant women can more easily experience specific micronutrient deficiencies such as a deficiency of vitamin B12, while school children or adolescents can more easily develop eating disorders. While plant-based diets offer numerous health benefits, it is important to carefully consider both the positive and negative aspects and to adopt a balanced approach to dietary choices. Further research is needed to fully understand the impacts of plant-based diets on the health and lifestyle habits of the population in order to provide more comprehensive information and support informed and healthy dietary choices.

## Figures and Tables

**Table 1:** Conversion table from qualitative questions into quantitative questions of the IASE index.

Conversion Table Applied to IASE					
Variables	Score				
	10	7.5	5	2.5	0
Fruit	1 piece/portion a day, 2–4 portions a day, ≥5 portions a day				Never or rarely
Vegetables	Every day	≥5 pieces per week, 2–4 pieces a week	1 piece/ration a week		Never or rarely
Cereals	Every day	≥3 times a week	1–2 times a week		Never or rarely
Milk	Every day	≥3 times a week	1–2 times a week		Never or rarely
Medium between white and red meat	1–2 times a week	≥3 times a week		Every day	Never or very rarely
Legumes	1–2 times a week	≥3 times a week		Every day	Never or very rarely
Soft drinks	Never or rarely	Very few times (2 times maximum per month)	One glass per week	≥2 glasses per week	2 glasses every day, 3–5 glasses every day, ≥5 glasses every day
Variety	2 points if each of the daily recommendations is met, 1 point if each of the weekly recommendations is met.				

**Table 2:** Categorization of the health and lifestyle variables and the variables for eating disorders.

Variable	Category	Score
Sleeping hours	Less than 6 h	1
	Between 6 and 7 h	2
	Between 7 and 8 h	3
	More than 8 h	4
Getting up rested	Never	1
	Very seldom and sometimes	2
	Frequently and almost always	3
	Always	4
Sleep quality	0 and 1	1
	2	2
	3	3
	4 and 5	4
Water	Never and very rarely (2 max. per month) and 1 glass/cup/week and ≥2 glasses/cups/week	1
	2 glasses/cups or less every day	2
	3–5 glasses every day	3
	More than 5 glasses every day	4
Sugary soft drinks, coffee, and energy drinks	Never and very rarely (2 glasses max. per month)	4
	One glass per week and 2 or more glasses per week	3
	2 glasses or less every day	2
	3–5 glasses and ≥5 glasses every day	1
Juice	Never and very rarely (2 glasses max. per month)	1
	One glass per week and 2 or more glasses per week	2
	2 glasses or less every day	3
	3–5 glasses and ≥5 glasses every day	4
Fish consumption	Never or very seldom	1
	1–2 times a week	2
	≥3 times a week	3
	Every day	4
Consumption of fast food, fried, and ultra-processed dishes	Never	1
	Very seldom (2 times a month maximum)	2
	Once a week	3
	Several times a week	4
Getting drunk	Never or less than once a month	1
	Monthly	2
	Weekly	3
	Daily or almost daily	4
Alcohol consumption	Never or once a month	1
	2–4 times a month	2
	2–3 times a week	3
	4–5 times a week or every day	4
Smoking	Non-smoker	1
	Light smoker (less than 5 cigarettes per day)	2
	Moderate smoker (6–15 cigarettes per day)	3
	Severe smoker (≥16 cigarettes per day)	4
Night outings	Never and sporadically	1
	1–2 times a week	2
	≥3 times a week	3
	Every day	4
Sedentary lifestyle	<7 h	1
	7–9 h	2
	9–11 h	3
	>11 h	4
Obesophobia, no control over eating intake, concern over body image	Never	1
	Rarely	2
	Occasionally	3
	Frequently	4
	Very frequently	5
	Always	6

**Table 3:** Sample and its socio-demographic characteristics (*n =* 19,211).

	Mean ± SD or *n* (%)
Male	3682 (19.17%)
Female	15,529 (80.83%)
Age (years)	34.49 ± 11.79
Male age	36.52 ± 13.72
Female age	34.02 ± 11.23
	**Total**
**Education level**	
Basic education	6050 (31.49%)
Higher education	13,161 (68.51%)
**Income level**	
Low	8370 (43.57%)
Medium–high	9220 (47.99%)
Do not know/no answer	1621 (8.44%)
**Municipality**	
<2000	865 (4.50%)
2000–10,000	3065 (15.95%)
>10,000	17,281 (79.54%)

**Table 4:** Diet prevalence according to socio-demographic groups, *n =* 19,211.

		Mediterranean Diet *n* (%)	Vegetarian *n* (%)	Vegan *n* (%)	Flexitarian *n* (%)	*p*-Value ^§^
**Sex**	Men	3467 (94.16%)	89 (2.42%)	50 (1.36%)	76 (2.06%)	0.85
	Women	14,106 (90.84%)	634 (4.08%)	315 (2.03%)	474 (3.05%)	
**Age**	18–25 years old	4829 (88.22%)	325 (5.94%)	134 (2.45%)	186 (3.40%)	0.23
	26–45 years old	9190 (91.57%)	352 (3.51%)	195 (1.94%)	299 (2.98%)	
	46–65 years old	3385 (95.92%)	46 (1.30%)	36 (1.02%)	62 (1.76%)	
	>65 years old	169 (98.26%)	0 (0%)	0 (0%)	3 (1.74%)	
**Education**	High education	12,045 (91.52%)	487 (3.70%)	238 (1.81%)	391 (2.97%)	1.00
	Low education	5528 (91.37%)	236 (3.90%)	127 (2.10%)	159 (2.63%)	
**Income**	Low income	7549 (90.19%)	356 (4.25%)	204 (2.44%)	261 (3.12%)	0.92
	Medium–high income	8549 (92.72%)	293 (3.12%)	132 (1.43%)	246 (2.67%)	
**Municipality**	Small city	796 (92.02%)	30 (3.47%)	20 (2.31%)	19 (2.20%)	1.00
	Medium city	2800 (91.35%)	121 (3.95%)	56 (1.83%)	88 (2.87%)	
	Big city	13,977 (91.47%)	572 (3.74%)	289 (1.89%)	443 (2.90%)	

**^§^** Chi-2 test.

**Table 5:** Distribution of nutritional, social, and lifestyle habit variables and health variables among sample groups according to the diet followed.

Numerical Variable	Mediterranean Diet (M)	Vegetarian Diet (VD)	Vegan Diet (VG)	Flexitarian Diet (FD)	*p*-Value *
BMI	23.96 ± 4.31	22.41 ± 3.57	22.56 ± 3.6	22.64 ± 3.58	*p* < 0.001 M-VD (*p* < 0.001) M-VG (*p* < 0.001) M-FD (*p* < 0.001)
IASE	54.86 ± 9.48	50.27 ± 6.55	41.81 ± 5.85	52.97 ± 7.53	*p* < 0.001 M-VD (*p* < 0.001) M-VG (*p* < 0.001) M-FD (*p* < 0.001) VD-VG (*p* < 0.001) VD-FD (*p* < 0.001) VG-FD (*p* < 0.001)
Fried food	2.33 ± 0.81	2.01 ± 0.77	1.96 ± 0.79	1.99 ± 0.72	*p* < 0.001 M-VD (*p* < 0.001) M-VG (*p* < 0.001) M-FD (*p* < 0.001)
Fast food	2.44 ± 0.75	2.31 ± 0.76	2.28 ± 0.76	2.18 ± 0.75	*p* < 0.001 M-VD (*p* < 0.001) M-VG (*p* < 0.001) M-FD (*p* < 0.001) VD-FD (0.01)
Ultra-processed food	2.39 ± 0.95	2.26 ± 0.91	2.13 ± 0.82	2.07 ± 0.88	*p* < 0.001 M-VD (*p* < 0.001) M-VG (*p* < 0.001) M-VD (*p* < 0.001) V-FD (*p* < 0.001)
Water	3.38 ± 0.64	3.42 ± 0.62	3.43 ± 0.60	3.49 ± 0.6	*p* < 0.001 M-VG (*p* < 0.001)
Sugary soft drinks	1.43 ± 0.69	1.24 ± 0.55	1.2 ± 0.48	1.19 ± 0.46	*p* < 0.001 M-VD (*p* < 0.001) M-VG (*p* < 0.001) M-FD (*p* < 0.001)
Juice	1.26 ± 0.55	1.20 ± 0.47	1.22 ± 0.52	1.17 ± 0.46	*p* < 0.001 M-FD (*p* < 0.001)
Coffee and energy drinks	1.72 ± 0.71	1.63 ± 0.65	1.56 ± 0.68	1.71 ± 0.66	*p* < 0.001 M-VD (0.01) M-VG (*p* < 0.001) VG-FD (*p* < 0.001)
Sedentary lifestyle	1.58 ± 0.84	1.60 ± 0.83	1.61 ± 0.87	1.66 ± 0.88	0.21 ^‡^
Self-perceived health	3.82 ± 0.82	3.88 ± 0.81	3.91 ± 0.92	3.89 ± 0.77	*p* < 0.001 M-VG (0.02)
Sport	144.98 ± 170.91	187.95 ± 177.32	213.99 ± 212.94	191.51 ± 180.2	*p* < 0.001 M-VD (*p* < 0.001) M-VG (*p* < 0.001) M-FD (*p* < 0.001)
Sleeping hours	2.51 ± 0.73	2.59 ± 0.72	2.55 ± 0.74	2.63 ± 0.70	*p* < 0.001 M-VD (*p* < 0.001) M-VG (*p* < 0.001)
Getting up rested	2.54 ± 0.58	2.55 ± 0.56	2.53 ± 0.63	2.53 ± 0.55	0.87 ^‡^
Sleep quality	3.39 ± 1.02	3.43 ± 1.01	3.38 ± 1.11	3.39 ± 0.98	0.68 ^‡^
Smoking	1.25 ± 0.66	1.13 ± 0.45	1.11 ± 0.39	1.11 ± 0.42	*p* < 0.001 M-VD (*p* < 0.001) M-VG (*p* < 0.001) M-FD (*p* < 0.001)
Alcohol	1.80 ± 0.89	1.58 ± 0.75	1.50 ± 0.75	1.60 ± 0.76	*p* < 0.001 M-VD (*p* < 0.001) M-VG (*p* < 0.001) M-FD (*p* < 0.001)
Getting drunk	1.06 ± 0.30	1.06 ± 0.26	1.02 ± 0.21	1.05 ± 0.25	0.04 M-VG (0.03)
Night outings	1.20 ± 0.44	1.19 ± 0.42	1.10 ± 0.32	1.19 ± 0.43	*p* < 0.001 M-VG (*p* < 0.001) VD-VG (*p* < 0.001) VG-F (0.02)
Obesophobia	3.42 ± 1.43	3.45 ± 1.42	3.40 ± 1.42	3.47 ± 1.38	0.75 ^‡^
No control	2.75 ± 1.29	2.82 ± 1.25	2.67 ± 1.34	2.83 ± 1.21	0.03 ^‡^
Body image	3.57 ± 1.28	3.68 ± 1.29	3.69 ± 1.31	3.68 ± 1.26	0.02 ^‡^
Diagnosed eating disorder	0.03 ± 0.16	0.07 ± 0.25	0.08 ± 0.27	0.05 ± 0.23	*p* < 0.001 M-VG (*p* < 0.001) VD-VG (*p* < 0.001) VG-FD (*p* < 0.001)

* Kruskal–Wallis test with Bonferroni correction (pairwise comparisons). ^‡^ No significant differences between groups.

**Figure A1:**
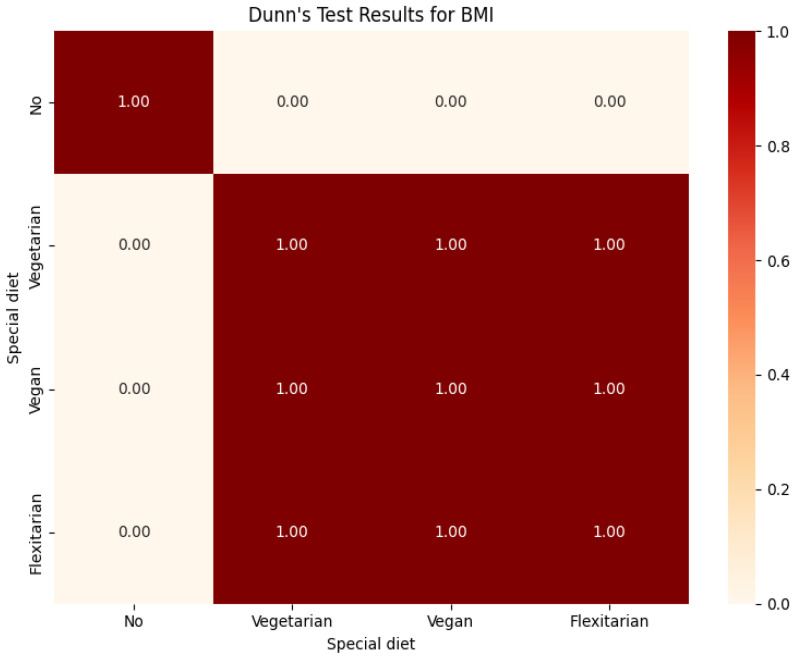
Dunn’s test results for BMI.

**Figure A2:**
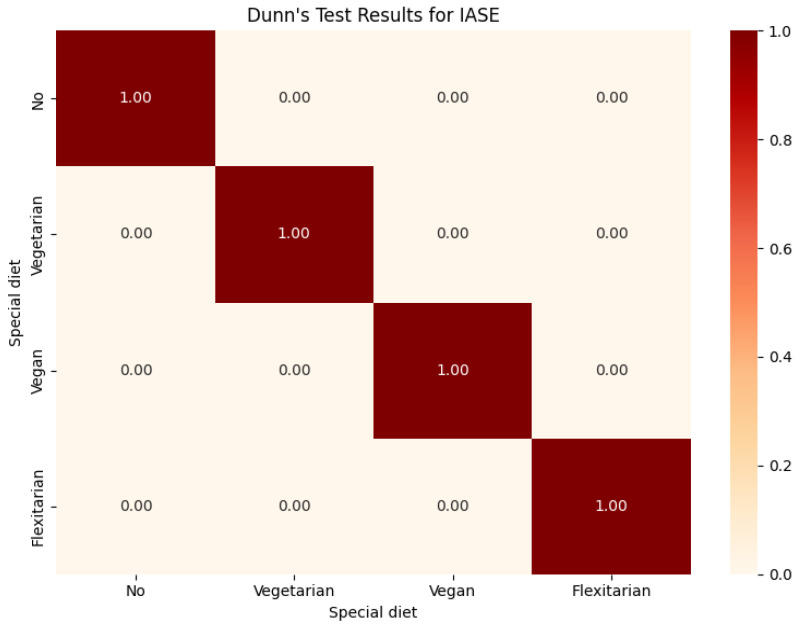
Dunn’s test results for IASE.

**Figure A3:**
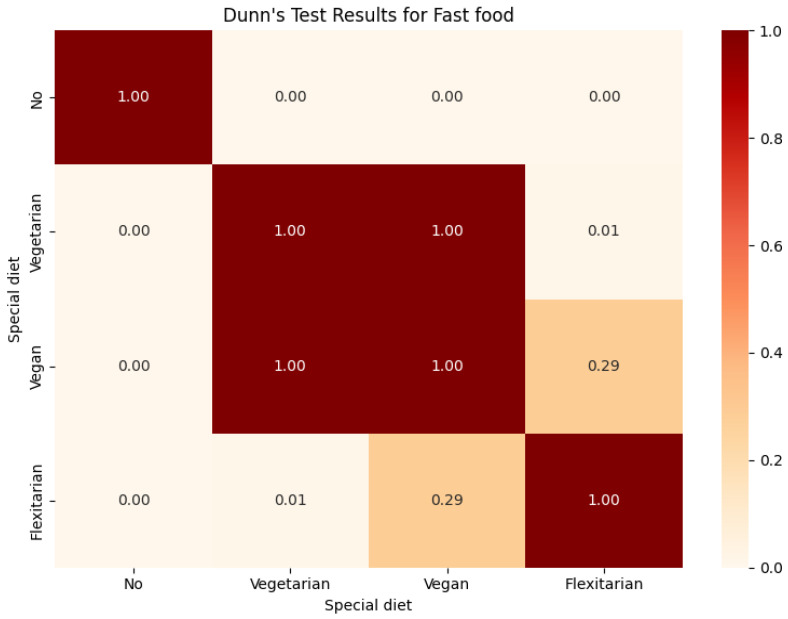
Dunn’s test results for fast food.

**Figure A4:**
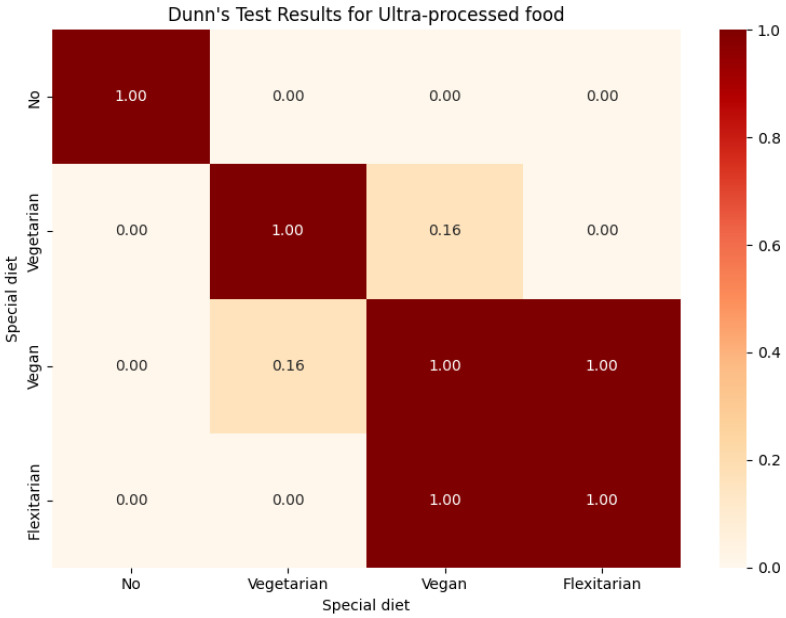
Dunn’s test results for ultra-processed food.

**Figure A5:**
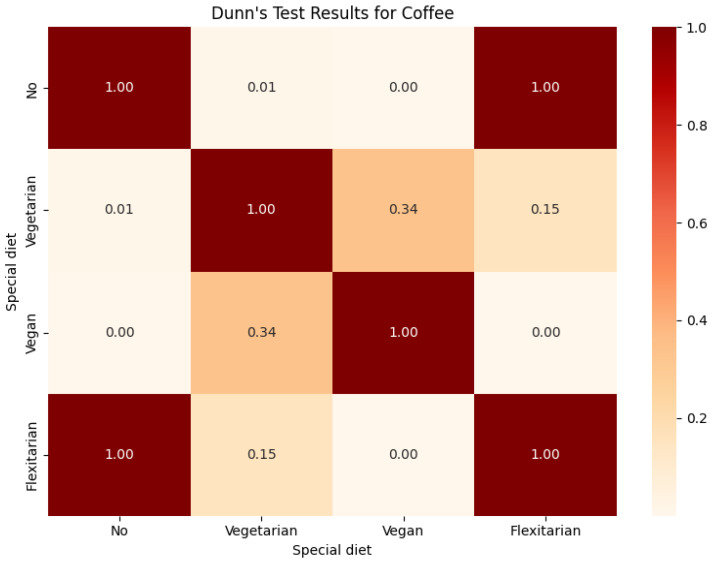
Dunn’s test results for coffee and energy drinks.

**Figure A6:**
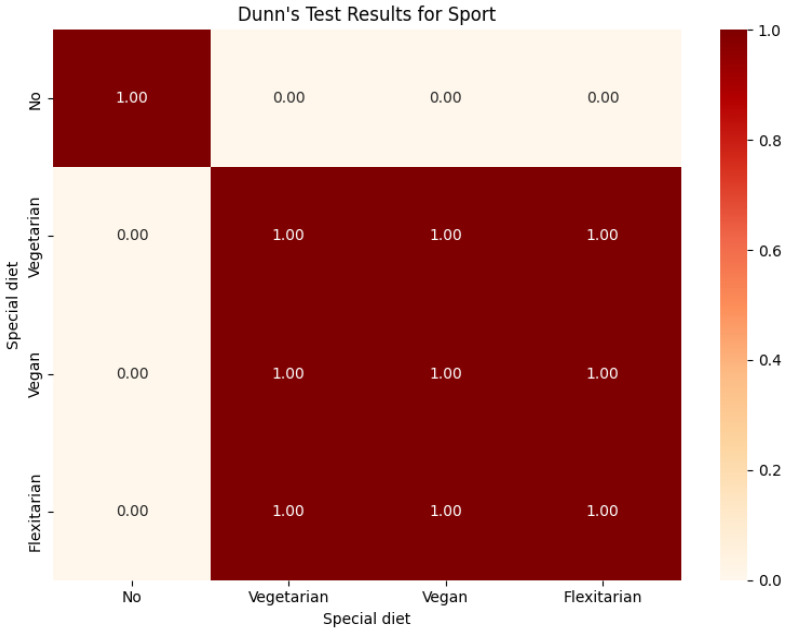
Dunn’s test results for sport.

**Figure A7:**
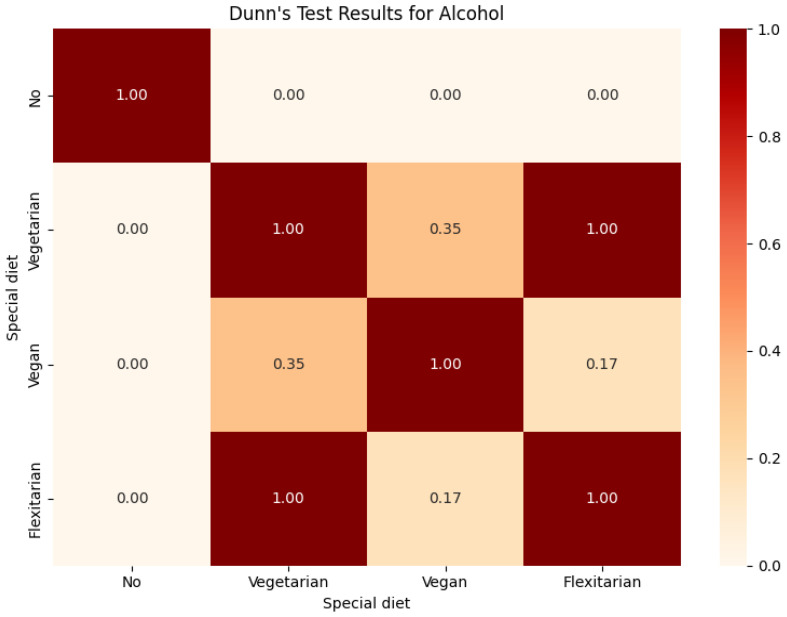
Dunn’s test results for alcohol.

**Figure A8:**
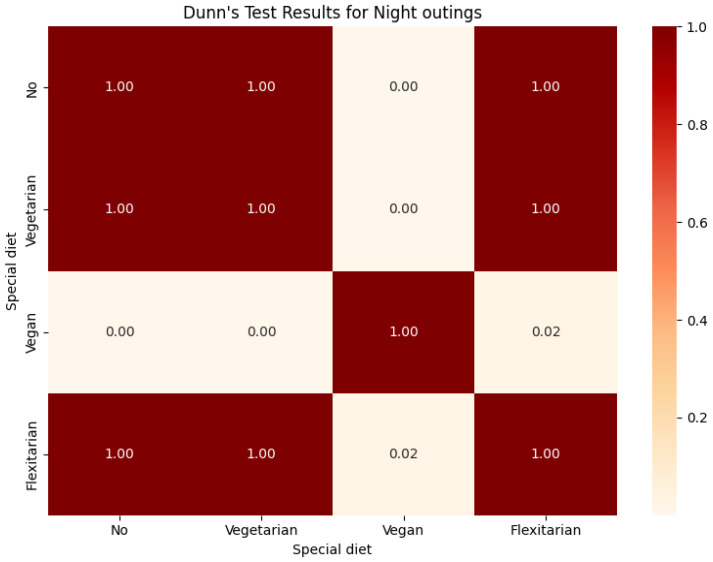
Dunn’s test results for night outings.

## Data Availability

The data presented in this study are available upon reasonable request from the corresponding author. The data are not publicly available due to privacy.

## Supplementary Materials

The following supporting information can be downloaded at: https://www.mdpi.com/article/10.3390/nu16091278/s1, STROBE checklist.

## Appendix A

Figure A1, Figure A2, Figure A3, Figure A4, Figure A5, Figure A6, Figure A7 and Figure A8 show the results of the Dunn’s Test performed for the most significant nutrition and lifestyle variables for the four population groups studied (people who followed a Mediterranean diet, respondents who adopted a vegan dietary pattern, those who preferred a vegetarian diet, and people who choose flexitarianism).
